# Thermodynamic Constraints on Electromicrobial Protein Production

**DOI:** 10.3389/fbioe.2022.820384

**Published:** 2022-02-21

**Authors:** Lucas Wise, Sabrina Marecos, Katie Randolph, Mohamed Hassan, Eric Nshimyumukiza, Jacob Strouse, Farshid Salimijazi, Buz Barstow

**Affiliations:** > ^1^ Department of Food Sciences, Cornell University, Ithaca, NY, United States; ^2^ Department of Biological and Environmental Engineering, Cornell University, Ithaca, NY, United States

**Keywords:** electromicrobial production, electron uptake, hydrogen oxidation, nitrogen fixation, carbon fixation

## Abstract

Global consumption of protein is projected to double by the middle of the 21st century. However, protein production is one of the most energy intensive and environmentally damaging parts of the food supply system today. Electromicrobial production technologies that combine renewable electricity and CO_2_-fixing microbial metabolism could dramatically increase the energy efficiency of commodity chemical production. Here we present a molecular-scale model that sets an upper limit on the performance of any organism performing electromicrobial protein production. We show that engineered microbes that fix CO_2_ and N_2_ using reducing equivalents produced by H_2_-oxidation or extracellular electron uptake could produce amino acids with energy inputs as low as 64 MJ kg^−1^, approximately one order of magnitude higher than any previous estimate of the efficiency of electromicrobial protein production. This work provides a roadmap for development of engineered microbes that could significantly expand access to proteins produced with a low environmental footprint.

## Introduction

### Current Methods of Protein Production Are Environmentally Damaging

Current food consumption and farming practices produce a large amount of environmental strain. In particular, the production of livestock for protein leads to significant waste accumulation and energy expenditure (McClements, 2019). The agricultural and food production sectors are responsible for ≈30% of greenhouse gas emissions, while livestock farming alone accounts for 18% of emissions (González et al., 2011). Furthermore, the agricultural industry is responsible for 70% of total freshwater consumption (Heinke et al., 2020). 42% of freshwater consumption is attributed to livestock production alone (Heinke et al., 2020). But, increased consumption of protein is one of the best ways to improve human, particularly infant, health and productivity in many parts of the world today (Ghosh et al., 2012).

The energy and water consumption of livestock farming will only increase as global appetites increase (Porritt and McCarthy, 2017). First, population will grow to ≈11 billion by 2050 (Prosekov and Ivanova, 2018). Second, the consumption of food, particularly protein, by each individual will also grow thanks to an expected average annual economic growth rate of 3% from 2014 to 2050 (Tilman et al., 2011; Hawksworth and Chan, 2015). Supplying this increased demand while maintaining the current agricultural areal footprint is expected to require a 75% increase in agricultural productivity (Prosekov and Ivanova, 2018).

Should agricultural production efficiencies remain stagnant, satisfying the food demands of the world’s growing and increasingly wealthy population with protein will require massive deforestation (Audsley et al., 2010; Tuomisto and Teixeira de Mattos, 2011). Deforestation could eradicate thousands of species and produce large quantities of greenhouse gases, leading to temperature increases exceeding the 2°C warming threshold established by the Paris Climate Agreement, even when ignoring emissions from all other human activity (Voegele and Nelson, 2019).

Incremental improvements in current food production technologies may not meet future demand and sustainability goals. Current approaches to increasing protein production include advanced livestock breeding, and substitution of livestock protein for insect- and plant-based substitutes. However, all of these approaches depend upon increases in crop yields. But, 78% of the world’s land has natural limitations for agricultural development (Prosekov and Ivanova, 2018), and significant doubts remain about the possibility of increasing crop yields by mid-century (Tilman et al., 2011; Slade et al., 2014; Poore and Nemecek, 2018). Furthermore, increasing water scarcity due to climate change could even depress crop yields in the decades ahead (Slade et al., 2014).

### Autotrophic Metabolism Could Increase the Efficiency of Protein Production

Autotrophic microbial production of protein is a promising alternative strategy to conventional food production (Ritala et al., 2017; Sillman et al., 2019; Mishra et al., 2020; Leger et al., 2021). In this class of schemes, externally supplied reducing equivalents are used to power microbial N_2_ and CO_2_-fixing metabolism and synthesis of protein molecules (Gleizer et al., 2020; Hu et al., 2020).

In most systems studied to date, reducing equivalents are supplied by H_2_^−^ or methane-oxidation. CO_2_-fixation is performed by Calvin-Benson-Bassham cycle, the reverse Krebs cycle or the Wood-Ljungdahl pathway.

Autotrophically produced protein has at least two important advantages over traditional protein production methods. Secondly, autotrophic protein production does not depend on the availability of arable land and can be run in a closed system. This greatly reduces water and land consumption and inhibits nitrogen runoff to surrounding environments (Sillman et al., 2019; Nyyssölä et al., 2021). Finally, autotrophic microorganisms can use atmospheric N_2_ as a substrate, eliminating the need for thermochemical N_2_-fixation (Bothe et al., 2010).

The cost of autotrophic protein production is dropping rapidly. The cost of production of a single protein has reduced from $1 × 10^6^ kg^−1^ in 2000 to ≈ $100 kg^−1^ in 2019 (Tubb and Seba, 2021). It is projected that the cost of production of a single protein could drop to below $10 kg^−1^ by 2025, thereby achieving price parity with animal-based protein products (Tubb and Seba, 2021).

Theoretical analysis suggests that autotrophic protein production could far exceed the efficiency of plant-based protein. Recent analyses of the performance of electromicrobial production of biofuels (Claassens et al., 2019; Salimijazi et al., 2019; Salimijazi et al., 2020), where electrically-supplied reducing equivalents are used to power CO_2_ fixation or formic acid assimilation and biofuel, show that these types of schemes could dramatically exceed the efficiency of photosynthetic biofuel production. These results imply that if N_2_ fixation were added to these systems, proteins could also be produced at efficiencies exceeding that of photosynthesis. Recent results by Leger et al. (2021) suggest photovoltaic-driven EMP of protein could exceed efficiency of real-world photosynthetic production of protein by at least 2 orders of magnitude.

However, up until now, very few attempts have been made at calculating the upper limit efficiency of EMP amino acid or protein production. This paper presents a model and analyzes the theoretical maximum energetic efficiency for a system of autotrophic microorganisms, fixing CO_2_ and N_2_ using electrons delivered by either extracellular electron uptake (EEU) (Rowe et al., 2021) or by H_2_-oxidation (Liu et al., 2016). These calculations do not predict the performance of any naturally-occurring organism, but do predict an upper limit efficiency for any natural or synthetic organism using these reactions.

## Theory, Results and Discussion

### Theory

We extended our theoretical framework for calculating the efficiency of electromicrobial production (EMP) of biofuels to calculate the efficiency of amino acid production from electrons, CO_2_ and N_2_ (Salimijazi et al., 2020). A full set of model parameters and associated values used in this article are shown in Table 1, and a full set of symbols for this article are shown in Supplementary Table S1.

**TABLE 1:** Electromicrobial protein production model parameters. Model parameters used in this article are based upon model parameters used in a previous analysis of the electromicrobial production of the biofuel butanol (Salimijazi et al., 2020). A sensitivity analysis was performed for all key parameters in this work (Salimijazi et al., 2020). A complete list of symbols used in this work (including symbols for outputs, and intermediate variables) is included in Supplementary Table S1.

Parameter	Symbol	1. H_2_	2. EEU	3. H_2_ with formate	4. EEU with formate
Electrochemical Cell Parameters					
Input solar power (W)	*P*_*γ*_	1,000	1,000	1,000	1,000
Total available electrical power (W)	*P*_e, total_	330	330	330	330
CO_2_-fixation method		Enzymatic		Electrochemical	
Electrode to microbe mediator		H_2_	EEU	H_2_	EEU
Cell 1 anode std. potential (V)	*U*_cell 1, anode, 0_	N/A		0.82 Torella et al., 2015	
Cell 1 anode bias voltage (V)	*U*_cell 1, anode, bias_	N/A		0.47 Liu et al., 2016	
Cell 1 anode voltage (V)	*U*_cell 1, anode_ = *U*_cell 1, anode, 0_ + *U*_cell 1, anode, bias_	N/A		1.29	
Cell 1 cathode std. potential (V)	*U*_cell 1, anode, 0_	N/A		−0.43 (Yishai, (2017), Zhang, (2018))	
Cell 1 cathode bias voltage (V)	*U*_cell 1, cathode, bias_	N/A		−1.3 (White et al., 2014)	
Cell 1 cathode voltage (V)	*U*_cell 1, cathode_ = *U*_cell 1, cathode, 0_ + *U*_cell 1, cathode, bias_	N/A		−1.73	
Cell 1 voltage (V)	Δ*U*_cell 1_ = *U*_cell 1, cathode_ − *U*_cell 1, anode_	N/A		3.02	
Cell 1 Faradaic efficiency	*ξ*_I1_	N/A		0.8 (Rasul et al., 2019)	
Carbons per primary fixation product	*ν*_Cr_	N/A		1	
*e* ^−^ per primary fixation product	*ν*_er_	N/A		2	
Cell 2 (Bio-cell) anode std. potential (V)	*U*_cell 2, anode, 0_	−0.41 (Torella et al., 2015)	−0.1 Bird, (2011), Firer-Sherwood, (2008)	−0.41 (Torella et al., 2015)	−0.1 (Bird, (2011), Firer-Sherwood, 2008)
Cell 2 (Bio-cell) anode bias voltage (V)	*U*_cell 2, anode, bias_	−0.3 (Liu et al., 2016)	−0.2 Ueki, (2018)	−0.3 Liu et al., 2016	−0.2 Ueki, (2018)
Cell 2 (Bio-cell) anode voltage (V) (**RCv2_1.02**)	*U*_cell 2, anode_ = *U*_cell 2, anode_, 0 + *U*_cell 2, anode, bias_	−0.71	−0.3	−0.71	−0.3
Cell (2) Bio-cell cathode std. potential (V)	*U*_cell 2, cathode, 0_	0.82			
Cell 2 (Bio-cell) cathode bias voltage (V)	*U*_cell 2, cathode, bias_	0.47			
Cell 2 (Bio-cell) cathode voltage (V)	*U*_cell 2, cathode_ = *U*_cell 2, cathode, 0_ + *U*_cell 2, cathode, bias_	1.29			
Bio-cell voltage (V)	Δ*U*_cell 2_ = *U*_cell 2, cathode_ − *U*_cell 2, anode_	2 (Liu et al., 2016)	1.59	2	1.59
Bio-cell Faradaic efficiency	*ξ*_I2_	1.0			
Cellular Electron Transport Parameters					
Membrane potential difference (mV)	Δ*U*_membrane_	140		140	
Terminal e- acceptor potential (V)	*U*_Acceptor_	0.82			
Quinone potential (V)	*U*_Q_	−0.0885 Bird, (2011)		−0.0885 Bird, (2011)	
Mtr EET complex potential (V)	*U*_Mtr_	N/A	−0.1 (Salimijazi et al., 2020)	N/A	−0.1 (Salimijazi et al., 2020)
No. protons pumped per *e* ^−^	*p*_out_	Unlimited		Unlimited	
Product Synthesis Parameters					
No. ATPs for product synthesis	*ν*_p, ATP_	See Supplementary Dataset S2			
No. NAD(P)H for product	*ν*_p, NADH_	See Supplementary Dataset S2			
No. Fdred for product	*ν*_p, Fd_	See Supplementary Dataset S2			
Product energy density (J molecule−1)	*E*_protein_	See Supplementary Table S2			

**FIGURE 1:**
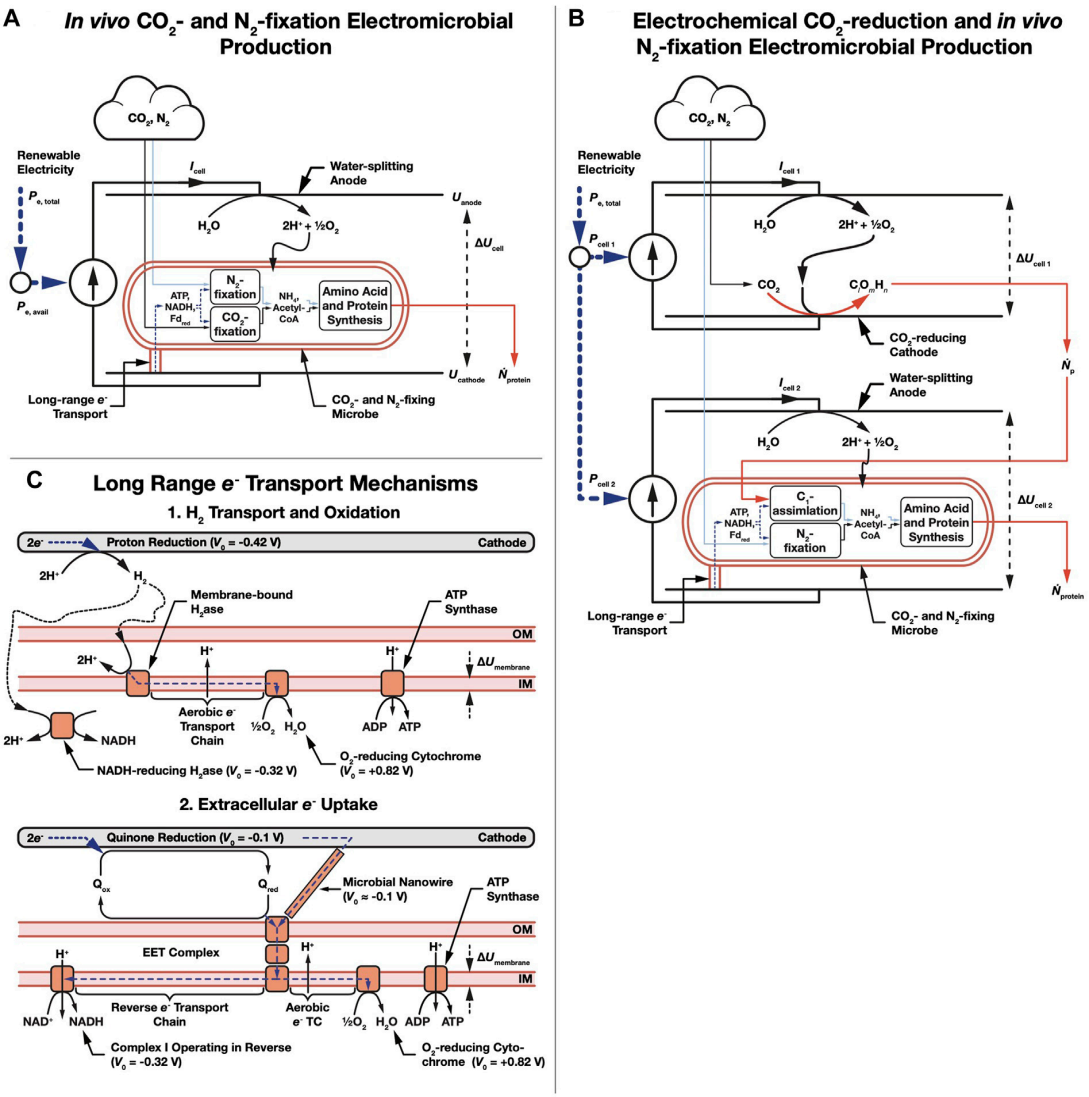
Schematic of amino acid electromicrobial production systems. **(A)** Single bio-electrochemical cell system where electricity is used to power *in vivo* CO_2_- and N_2_-fixation. **(B)** Dual electrochemical cell system where CO_2_ is reduced in the first cell, and then assimilated in the second cell, and combined with enzymatically fixed N_2_. **(C)** Long range e- transfer mechanisms considered in this article. In the first, H_2_ is electrochemically reduced on a cathode, transferred to the microbe by diffusion or stirring, and is enzymatically oxidized. In the second mechanism, extracellular electron uptake (EEU), e- are transferred along a microbial nanowire (part of a conductive biofilm), or by a reduced medium potential redox shuttle like a quinone or flavin, and are received at the cell surface by the extracellular electron transfer (EET) complex. From the thermodynamic perspective considered in this article, these mechanisms are equivalent. Electrons are then transported to the inner membrane where reverse electron transport is used to regenerate NAD(P)H, reduced Ferredoxin (not shown), and ATP (Rowe et al., 2018; Rowe et al., 2021). Note that we use the American and British classical current convention where current flows from positive to negative.

We consider a bio-electrochemical system used to deliver electrons to microbial metabolism (Figures 1A,B). Electrical power is used to generate amino acid (or protein) molecules with an energy per molecule *E*_protein_ at a rate *Ṅ*_protein_. Even though this article strictly considers amino acid synthesis, this can be considered equivalent to protein production from an energetic standpoint as no energy is expended in forming the peptide bond needed to polymerize amino acids. We choose to use the subscript protein rather than AA to avoid confusion with the Avogadro constant, *N*_A_. Energy per molecule and molecular weight for each amino acid are shown in Supplementary Table S2. Full derivations of the equations presented here can be found in the supplement to our original electromicrobial production efficiency theory article (Salimijazi et al., 2020), with some changes of symbols used to indicate that we are producing proteins rather than amino acids. If a change of symbol is used, it is indicated in Supplementary Table S1. In our original article (Salimijazi et al., 2020) we focused purely on electrical (or solar) energy to chemical energy (fuel, or on this case protein) conversion efficiency, but in this article we expand our theory to calculate the energy (electrical or solar) costs of producing a gram of product (*C*_EP_ and *C*_SP_, respectively).

The energy conversion efficiency of the system from electricity to amino acids (or protein) is calculated from the ratio of the amount of chemical energy stored per second (*Ṅ*_protein_ *E*_protein_), relative to the power input to the system, *P*_e,total_ (Salimijazi et al., 2020) (basically power out to power in),

$$\eta_{EP}={\overset{\cdot }{N}}_{protein}E_{protein}/P_{e,total}.$$ (1)

The total mass of protein produced per second by the system is,

$${\overset{\cdot }{m}}_{protein}={\overset{\cdot }{N}}_{protein}M_{protein}/N_{A}.$$ (2)

where *M*_protein_ is the molecular weight of the protein molecule.

The energy cost to produce a unit mass of protein,

$$C_{EP}=P_{e,\;total}N_{A}/\left({\overset{\cdot }{m}}_{protein}\right),$$ (3)

Thus, if both the chemical energy per protein molecule and the molecular weight are known (they are for proteins), energy conversion efficiency and energy cost can be easily interconverted,

$$C_{EP}=N_{A}E_{protein}/\left(\eta_{EP}M_{protein}\right),$$ (4)

thus,

$$C_{EP}=P_{e,total}N_{A}/\left({\overset{\cdot }{N}}_{protein}M_{protein}\right),$$ (5)

For a single bio-electrochemical cell system where CO_2_- and N_2_- fixation are performed *in vivo* (Figure 1A), the upper limit electrical to chemical conversion efficiency of the system is set by the energy density of an amino acid molecule relative to the amount of charge needed to synthesize it from CO_2_ and N_2_ (the fundamental charge, *e*, multiplied by the number of electrons needed for synthesis, *ν*_ep_) and the potential difference across the bio-electrochemical cell, Δ*U*_cell_,

$$\eta_{EP}\leq E_{protein}/\left(e\nu_{ep}\Delta U_{cell}\right).$$ (6)

Thus, the amount of electricity needed to produce a unit-mass of the protein is,

$$C_{EP}\geq N_{A}\Delta U_{cell}e\nu_{ep}/M_{protein}.$$ (7)

A full derivation of Eqs 1, 6 in this article can be found in Section 1 (Eqs 1−9) in the supplement of Salimijazi et al. (2020).

We also consider systems CO_2_ reduction is performed electrochemically, and the resulting reduction product (typically a C_1_ compound like formic acid) (White et al., 2014; White et al., 2015; Appel et al., 2013) is further reduced enzymatically (Figure 1B). While a C1 compound like formic acid is lightly reduced (e.g., 2 *e* ^−^ per carbon for formic acid), carbohydrates, biofuels, a protein molecules are more heavily reduced (4–6 *e* ^−^ per carbon). Thus, while formic acid can supply all of the carbon and some of the electrons needed to make a protein, it cannot supply all of the electrons, and these need to be supplied by EEU or H_2_-oxidation. In these cases, *ν*_ep_ is substituted for the number of additional electrons needed to convert the C_1_ product into the final protein product, *ν*_e,add_ (Salimijazi et al., 2020),

$$\eta_{EP}\leq \frac{E_{protein}\xi_{I2}}{e\nu_{e,add}\left(\Delta U_{cell1}\left(\frac{\nu_{r}\nu_{er}\nu_{Cr}\xi_{I2}}{\xi_{I1}\xi_{C}\nu_{e,add}}\right)+\Delta U_{cell2}\right)},$$ (8)

where *ν*_r_ is the number of primary reduction products (i.e., formic acid molecules) needed to synthesize a molecule of the final product, *ν*_er_ is the number of electrons needed to reduce CO_2_ to a primary reduction product (i.e., 2 in the case of formic acid), *ν*_Cr_ is the number of carbon atoms per primary fixation product (i.e., 1 in the case of formic acid), *ξ*_I2_ is the Faradaic efficiency of the bio-electrochemical cell, *ξ*_I1_ is the Faradaic efficiency of the primary abiotic cell 1, *ξ*_C_ is the carbon transfer efficiency from cell 1 to cell 2. A full derivation of Eq. 8 can be found in Section 10 (Equations 101–118) of the supplement of Salimijazi et al. (2020).

Thus, using Eq. 4, the amount of electricity needed to produce a unit-mass of the protein when using electrochemical CO_2_-reduction is,

$$C_{EP}\geq \frac{e\nu_{e,add}N_{A}\left(\Delta U_{cell1}\left(\frac{\nu_{r}\nu_{er}\nu_{Cr}\xi_{I2}}{\xi_{I1}\xi_{C}\nu_{e,add}}\right)+\Delta U_{cell2}\right)}{M_{protein}\xi_{I2}}.$$ (9)

We calculate the electron requirements, *ν*_ep_ or *ν*_e,add_, for amino acid (or protein) synthesis from the number of NAD(P)H (*ν*_p,NADH_) reduced Ferredoxin (Fd_red_; *ν*_p,Fd_) and ATP (*ν*_p,ATP_) molecules needed for the synthesis of the molecule, along with a model of the mechanism used for electron delivery to the microbe (Salimijazi et al., 2020).

The key part of our electromicrobial production efficiency theory (Salimijazi et al., 2020) answers the question: how efficiently can energy carried by H_2_ or by EEU be transferred into the intracellular reductants needed for metabolism (ATP, NAD(P)H, and Ferredoxin) by use of the inner membrane proton gradient. In the case of both H_2_-oxidation (autotrophic growth of *Ralstonia eutropha*, the organism used in the Bionic Leaf (Liu et al., 2016), typically uses an atmospheric ratio of 8:1:1 H_2_:O_2_:CO_2_ (Brigham et al., 2013)) and EEU (Rowe et al., 2018; Salimijazi et al., 2020; Rowe et al., 2021) mediated electromicrobial production, a micro-aerobic atmosphere needs to be maintained in the cathode chamber. The O_2_ concentration in the cathode chamber needs to be just high enough to provide a terminal electron acceptor capable of generating the most proton motive force per electron input into the system, yet low enough to not be reduced by the cathode to H_2_O and short-circuit the electrochemical system. It is notable that both the anode and cathode in the Bionic Leaf exist in the same reaction chamber suggesting that a small amount of O_2_ is constantly present. Despite this, the energy efficiency (and by extension Faradaic efficiency) is remarkably high (Liu et al., 2016) (**RCv2_1.01**).

For systems that rely upon H_2_-oxidation for electron delivery like the Bionic Leaf (Torella et al., 2015; Liu et al., 2016; Salimijazi et al., 2020) (Figure 1C, part 1), the number of electrons needed to synthesize one amino acid molecule is,

$$\nu_{ep,H_{2}}=2\nu_{p,NADH}+2\nu_{p,Fd}+\nu_{p,ATP}\frac{ceil\left(\Delta G_{ATP/ADP}/e\Delta U_{membrane}\right)}{floor\left(\left(U_{H_{2}}-U_{acceptor}\right)/\Delta U_{membrane}\right)},$$ (10)

where Δ*G*_ATP/ADP_ is the free energy required for regeneration of ATP, Δ*U*_membrane_ is the potential difference across the cell’s inner membrane due to the proton gradient, *U*_H2_ is the standard potential of proton reduction to H_2_, *U*_acceptor_ is the standard potential of terminal electron acceptor reduction (typically O_2_ + 2*e* ^−^ to H_2_O), the ceil function rounds up the nearest integer, and the floor function rounds down to the nearest integer. A full derivation of Eq. (10) can be found in Section 2 (Equations 10 to 20) of the supplement for Salimijazi et al. (2020).

The first and second terms in Eq. 10 describe the number of electrons needed to regenerate the NAD(P)H and Ferredoxin needed for amino acid synthesis. As the redox potential of H_2_ is above those of both NADH and Ferredoxin and both molecules require two electrons to be regenerated, two electrons can be transferred directly from H_2_-oxidation. Thus, the number of electrons needed for NAD(P)H regeneration is just double the number of NAD(P)H and Ferredoxin needed for synthesis of the amino acid.

The final term in Eq. 10 calculates the number of electrons needed to regenerate the ATP needed for amino acid synthesis. ATP regeneration involves energy transfer from the incoming electrons to ATP, and charge transfer to O_2_. The numerator in the final term of Eq. 10 calculates the number of protons that need to be pumped through the ATP synthase in order to regenerate 1 ATP: the energy needed to regenerate 1 ATP divided by the energy recovered by pumping one proton from the periplasmic side of the inner membrane to the cytoplasmic side. As only integral numbers of protons can be pumped, the ceil function rounds up the result. The denominator in the final term of Eq. 10 calculates how many protons can be pumped from the cytoplasmic side of the membrane to the by sending 1 electron downhill from H_2_ to the acceptor (O_2_/H_2_O). Again, as only integral numbers of protons can be pumped, the floor function rounds down.

The appearance of Δ*U*_membrane_ in the numerator and denominator of Eq. 10 is required because the ceil and floor functions are numerical (not analytical) and require their arguments to be numerically evaluated before the result can be used in a larger calculation. This is initially counter-intuitive, but captures the core of the unavoidable energy losses imposed by using proton pumping to transduce energy. To illustrate this, consider this example: the result of 7/2 divided by 5/2 is just 7/5 or 1.4 (the twos in the denominators of both terms cancel). However, the result of ceil (7/2) divided by floor (5/2) is different: ceil (7/2) is ceil (3.5) or 4, while floor (5/2) is floor (2.5) or 2. Thus ceil (7/2) divided by floor (5/2) is 2.0, 43% higher than the result of 7/5.

**FIGURE 2:**
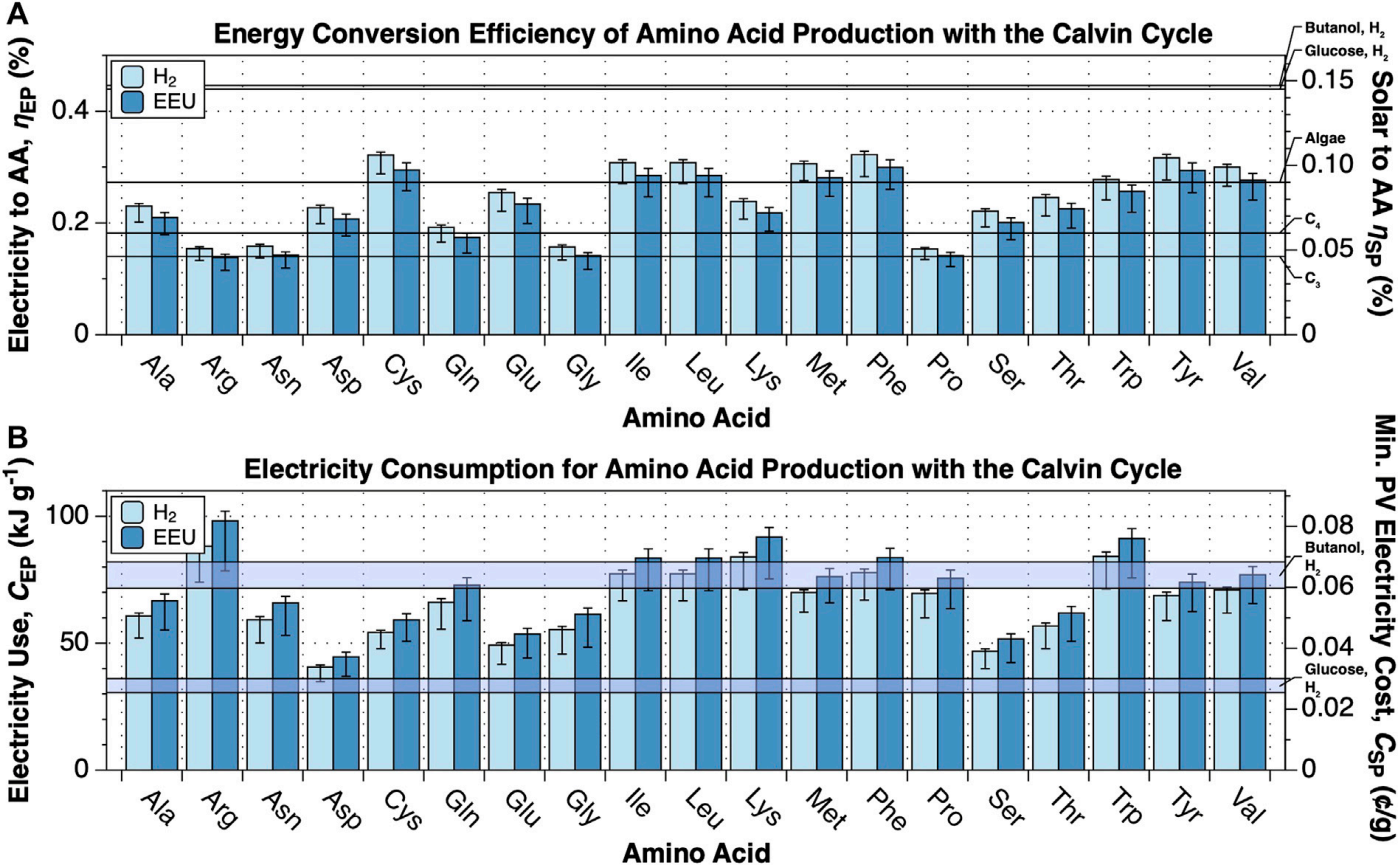
Energy conversion efficiency and energy cost of amino acid production. The upper limit energy conversion efficiency and minimum energy cost of amino acid production from CO_2_, N_2_ and electricity by electromicrobial production systems using the Calvin cycle for CO_2_-fixation and either H_2_-oxidation or extracellular electron uptake (EEU) were calculated for 19 dietary amino acids (all except histidine) with the electrofoods package (Barstow, 2021). NADH, Fdred, and ATP requirements for synthesis of each amino acid are tabulated in Supplementary Dataset S2. This plot can be reproduced using the fig-cbb_n2_to_amino_acids.py program in the electrofoods package (Barstow, 2021). **(A)** Upper limit electrical and solar energy conversion efficiency for amino acids. The left axis shows the electricity to amino acid energy conversion efficiency, while the right axis shows the solar to amino acid conversion efficiency, assuming the system is supplied by a perfectly efficient single-junction Si solar photovoltaic (solar to electrical efficiency of 32.9% (Nelson, 2003)). As a first point of comparison, the upper limit solar to biomass energy conversion efficiencies of C3, C4 (Zhu et al., 2008; Zhu et al., 2010), and algal photosynthesis (Wijffels and Barbosa, 2010) are marked on the right axis. As a second point of comparison, we have also marked the projected upper limit solar to butanol (Salimijazi et al., 2020) and glucose (calculated here) conversion efficiencies by an electromicrobial production system using H2-oxidation and the Calvin cycle. **(B)** Minimum electrical and solar energy costs for the production of a gram of amino acids. The left axis shows the minimum electricity cost, while the right axis shows the minimum cost of that solar electricity, assuming that the United States Department of Energy’s cost target of 3 ¢ per kWh by 2030 can be achieved (United States Department of Energy, 2016).

**FIGURE 3:**
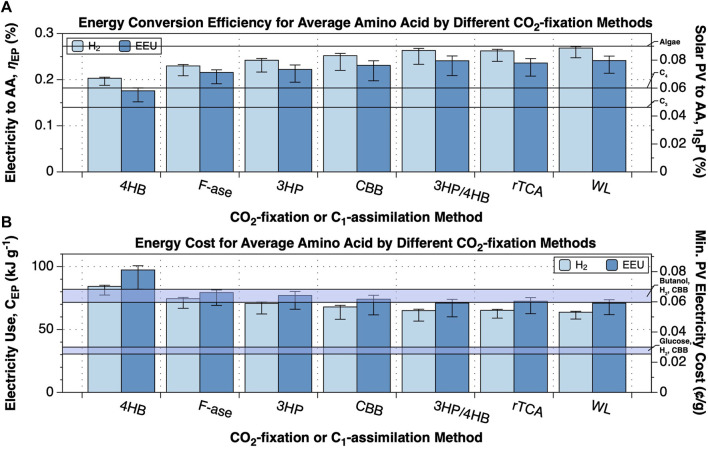
Changing CO_2_-fixation method can improve the performance of amino acid synthesis. The upper limit energy conversion efficiency and minimum energy cost of production of an average amino acid from CO_2_ or HCOO-, N_2_ and electricity by electromicrobial production systems using either H_2_-oxidation or extracellular electron uptake (EEU) and one of the 6 naturally-occurring CO_2_-fixation pathways or the synthetic Formolase formate assimilation pathway were calculated with the electrofoods package (Barstow, 2021). NADH, Fdred, and ATP requirements for synthesis of an average amino acid are tabulated in Supplementary Dataset S2. This plot can be reproduced using the fig-cbb_n2_to_amino_acids.py program in the electrofoods package (Barstow, 2021). 3HP, 3-hydroxypropionate cycle; 3HP-4HB, 3-hydroxypropionate/4-hydroxybutyate cycle; 4HB, 4-hydroxybutyate cycle; CBB, Calvin-Bensson-Bassham cycle; Form, Formolase pathway; rTCA, reductive TCA cycle; WL, Wood-Ljungdahl pathway. **(A)** Upper limit electrical and solar energy conversion efficiency for an average amino acid. The left axis shows the electricity to amino acid energy conversion efficiency, while the right axis shows the solar to amino acid conversion efficiency, assuming the system is supplied by a perfectly efficient single-junction Si solar photovoltaic (solar to electrical efficiency of 32.9% (Nelson, 2003)). **(B)** Minimum electrical and solar energy costs for the production of a gram of an average amino acid. The left axis shows the minimum electricity cost, while the right axis shows the minimum cost of that solar electricity, assuming that the United States Department of Energy’s cost target of 3 ¢ per kWh by 2030 can be achieved (United States Department of Energy, 2016).

The inner membrane potential difference, Δ*U*_membrane_, is the largest source of uncertainty in this calculation. Therefore, we present a range of efficiency estimates in Figures 2, 3 and throughout the text for Δ*U*_membrane_ = 80 mV (BioNumber ID (BNID) 10408284 (Milo et al., 2010)) to 270 mV (BNID 107135), with a central value of 140 mV (BNIDs 109774, 103386, and 109775).

For systems that rely upon EEU for electron delivery like *Shewanella oneidensis* (Salimijazi et al., 2020; Rowe et al., 2021) (Figure 1C, part 2),

$$\nu_{ep,EEU}=2\nu_{p,NADH}+2\nu_{p,Fd}+\nu_{p,ATP}\frac{ceil\left(\Delta G_{ATP/ADP}/e\Delta U_{membrane}\right)}{floor\left(\left(U_{Q}-U_{acceptor}\right)/\Delta U_{membrane}\right)}+\nu_{p,NADH}\frac{ceil\left(\left(U_{NADH}-U_{Q}\right)/\Delta U_{membrane}\right)}{floor\left(\left(U_{Q}-U_{acceptor}\right)/\Delta U_{membrane}\right)}+\nu_{p,Fd}\frac{ceil\left(\left(U_{Fd}-U_{Q}\right)/\Delta U_{membrane}\right)}{floor\left(\left(U_{Q}-U_{acceptor}\right)/\Delta U_{membrane}\right)},$$ (11)

where *U*_Q_ is the redox potential of the inner membrane electron carrier, thought to be ubiquinone (Rowe et al., 2018), *U*_NADH_ is the standard potential of NAD(P)H, and *U*_Fd_ is the standard potential of Ferredoxin. A full derivation of Eq. (11) can be found in Section 7 (Equations 77–91) of the supplement for Salimijazi et al. (2020).

Understanding the division of electron flow between proton motive force generation and electron carrier reduction within the EMP organism will allow us to estimate how low the O_2_ concentration can be driven.

The overall anode and cathode reactions for H_2_ evolution,

$$H_{2}O\to \frac{1}{2}O_{2}+H_{2}.$$ (12)

Thus for every two H_2_ molecules that are generated, one O_2_ molecule is also generated.

$$\nu_{O_{2}evolved,H_{2}}=\nu_{e,p,H_{2}}/4.$$ (13)

Likewise, for an EEU-mediated system,

$$\nu_{O_{2}evolved,EEU}=\nu_{e,p,EEU}/4.$$ (14)

How much of this O_2_ is actually needed by the microbe in order to use the H_2_ to generate protein molecules? The redox reaction carried out by complex IV, the terminal oxidase in the aerobic electron transport chain reduces O_2_ to water and transports (in net) 4 protons to the periplasmic (p) side of the inner membrane from the cytoplasm (cyt),

$$O_{2}+8H_{cyt}^{+}+4e_{p}^{-}\to 2H_{2}O+4H_{p}^{+}$$ (15)

Thus, one O2 molecule is consumed for every 4 electrons sent downhill in energy. Therefore, (from Eq. 10), the number of O_2_ molecules needed for H_2_-mediated EMP is just ^1^/_4_ of the number of electrons used to generate the proton motive force needed regenerate ATP,

$$\nu_{O_{2}consumed,H_{2}}=\frac{\nu_{p,ATP}}{4}\frac{ceil\left(\Delta G_{ATP/ADP}/e\Delta U_{membrane}\right)}{floor\left(\left(U_{H_{2}}-U_{acceptor}\right)/\Delta U_{membrane}\right)}.$$ (16)

Likewise, for EEU-mediated EMP, the number of O_2_ molecules needed is ^1^/_4_ of the number electrons used to generate the proton motive force needed to regenerate ATP, NAD(P)H and Ferredoxin (but not directly reduce NAD (PH) or Ferredoxin) (from Eq. 11),

$$\nu_{O_{2}consumed,H2}=\frac{\nu_{p,ATP}}{4}\frac{ceil\left(\Delta G_{ATP/ADP}/e\Delta U_{membrane}\right)}{floor\left(\left(U_{Q}-U_{acceptor}\right)/\Delta U_{membrane}\right)}+\frac{\nu_{p,NADH}}{4}\frac{ceil\left(\left(U_{NADH}-U_{Q}\right)/\Delta U_{membrane}\right)}{floor\left(\left(U_{Q}-U_{acceptor}\right)/\Delta U_{membrane}\right)}+\frac{\nu_{p,Fd}}{4}\frac{ceil\left(\left(U_{Fd}-U_{Q}\right)/\Delta U_{membrane}\right)}{floor\left(\left(U_{Q}-U_{acceptor}\right)/\Delta U_{membrane}\right)}.$$ (17)

The results of Eqs 13–17 are computed by the cbb_glycine_O2.py code in the electrofoods package (**RCv2_1.01**).

The NAD(P)H, ATP and Fd_red_ requirements for amino acid synthesis were calculated by balancing networks of reactions for the autotrophic synthesis of the molecule from N_2_ and CO_2_ or N_2_ and formate (COOH^−^). We enumerated all reaction steps for the production of 19 of the 20 dietary amino acids from acetyl-CoA and NH_4_ using data from the KEGG database in Supplementary Dataset S3 (Kanehisa and Goto, 2000; Kanehisa, 2019; Kanehisa et al., 2020). Synthesis of histidine was excluded from these calculations because of technical challenges with stoichiometric balancing due to its inseparable connection with purine synthesis. As a comparison point, and to validate our approach, we also consider the synthesis of glucose.

Amino acid synthesis reactions were complemented with reactions for CO_2_-fixation, C_1_-assimilation, and N_2_ fixation (Supplementary Table S3). For this article we considered 6 scenarios in which CO_2_ was fixed by the well-known Calvin cycle (Berg et al., 2002), the Reductive Tricarboxylic Acid cycle (Alissandratos and Easton, 2015; Claassens et al., 2016), Wood-Ljungdahl (WL) Pathway (Berg et al., 2002); the 3-hydroxypropionate/4-hydroxybutyrate (3HP-4HB) Pathway (Berg et al., 2007; Claassens et al., 2016); 3-hydroxypropionate (3HP) Cycle (Zarzycki et al., 2009); and the Dicarboxylate/4-hydroxybutyrate (4HB) Cycle (Huber et al., 2008). In addition, we also considered the artificial Formolase formate assimilation pathway (Siegel et al., 2015). Finally, in all scenarios, N_2_ was fixed into metabolism by the iron-molybdenum (FeMo) nitrogenase (Kyoto Encyclopaedia of Genes and Genomes (KEGG) reaction R05185 (Bothe et al., 2010; Hu et al., 2020; Nyyssölä et al., 2021).

The overall stoichiometry of autotrophic amino acid synthesis was calculated by a custom flux balance code. Amino acid synthesis reactions (Supplementary Dataset S1) were combined automatically with the CO_2_-fixation, C_1_-assimilation, and N_2_ fixation reactions (Supplementary Table S3) by a custom code (Barstow, 2021) into a set of stoichiometric matrices, **S**_**p**_, for each reaction network.

Each automatically generated stoichiometric matrix was balanced with a custom flux balance program (Barstow, 2021) to find the overall number of NAD(P)H, Fd_red_, and ATP needed for synthesis of each amino acid using each CO_2_-fixation or C_1_-assimilation pathway.

We consider a species number rate of change vector, ***ṅ***, that encodes the rate of change of number of the reactant molecules over a single cycle of the reaction network; a stoichiometric matrix **S**_**p**_ that encodes the number of reactants made or consumed in every reaction in the network; and a flux vector ***v*** that encodes the number of times each reaction is used in the network. Reactant molecules are denoted as inputs (e.g., CO_2_, N_2_, COOH^−^, ATP, NAD(P)H), outputs (e.g., H_2_O), intermediates, or the target molecule (e.g., the amino acid to be synthesized). For the purposes of this thermodynamic analysis, we consider NADH and NADPH to be equivalent as they have near identical redox potentials.

The reactant number vector elements for the inputs were calculated by numerically solving the flux balance equation,

$$\overset{\cdot }{n}=S_{p}v,$$ (18)

under the constraint that number of each intermediate does not change over a reaction cycle, and that number of target molecules increases by 1,

$${\overset{\cdot }{n}}_{i}=\{\begin{array}{cc} 0 & \;if\;species\;i\;is\;an\;\mathrm{int}ermediate\\ 1 & if\;species\;i\;is\;the\;t\arg et \end{array}.$$ (19)

The balanced overall stoichiometry for synthesis of each amino acid is shown in Supplementary Dataset S2.

The number of electrons needed to synthesize an average amino acid was found by calculating the average number of NAD(P)H, Fd_red_, and ATP needed for synthesizing 19 of the 20 amino acids.

## Results and Discussion

### Electromicrobial Production of Amino Acids and Protein

The electrical and solar energy to protein conversion efficiency (*η*_EP_ and *η*_*S*P_) and the electrical energy consumption per unit mass (*C*_EP_) and cost of solar electricity per unit mass (*C*_SP_) for the production of 19 amino acids was calculated for electron uptake by H_2_ transport and oxidation and EEU, and CO_2_ fixation by the Calvin cycle (Figure 2).

Amino acid synthesis has a lower conversion efficiency than purely carbon-containing products due to the high Fd_red_ and ATP requirements of N_2_-fixation (Supplementary Dataset S1). Despite this, the conversion efficiency either matches, and in most cases exceeds the theoretical maximum conversion efficiency of sunlight to carbohydrate biomass by C_3_ photosynthesis (Figure 2A). However, Arg, Asn, Gly, and Pro synthesis by H_2_ and EEU, and Gln synthesis by EEU have lower conversion efficiencies than C_4_ carbohydrate photosynthesis (Zhu et al., 2008; Zhu et al., 2010). Synthesis of Cys, Ile, Leu, Met, Phe, Tyr and Val exceed the theoretical efficiency of algal photosynthesis (Wijffels and Barbosa, 2010). The average CO_2_, N_2_, and electricity conversion efficiency for an average amino acid using the Calvin cycle is $25.2_{-3.2}^{+0.5}\%$ when using H_2_-oxidation, and $23.1_{-3.3}^{+1.0}\%$ when using EEU (Figure 3A).

The electrical energy costs (*C*_EP_) for individual amino acids using H_2_-oxidation an the Calvin cycle range from $40.6_{-5.8}^{+0.8}kJg^{-1}$ for Asp to $88.2_{-14.1}^{+1.9}kJg^{-1}$ for Arg (Figure 2B). Synthesizing the amino acids by EEU rather than H_2_ adds between ≈5 and 10 kJ g^−1^. At projected 2030 prices for solar photovoltaic electricity from the DOE’s SunShot program of 3 ¢ per kWh (United States Department of Energy, 2016), this corresponds to a minimum cost of 0.033 to 0.081 ¢ g^−1^ (Figure 2B). The average amino acid synthesis energy cost using H_2_-oxidation and the Calvin cycle is $67.9_{-9.8}^{+1.3}kJg^{-1}$ (Figure 3B).

As noted before, the energy conversion efficiency of systems using EEU is consistently a few percentage points lower than for systems using H_2_ oxidation (Salimijazi et al., 2020) (Figure 2A). In EEU based systems there is a higher electron requirement, and hence cell current, needed for regeneration of NAD(P)H, Fd_red_ and ATP. Practically, this is almost offset by a lower minimum cell voltage, resulting in a slightly lower conversion efficiency (Salimijazi et al., 2020). Averaged across all amino acids, the efficiency of synthesis for systems using EEU and the Calvin cycle is $23.1_{-3.3}^{+1.0}\%$ . This results in an average electrical energy cost that of $74.1_{-12.5}^{+3.1}kJg^{-1}$ , about $6kJg^{-1}$ higher than the cost of synthesis using H_2_-oxidation.

Can we increase the efficiency of electromicrobial production of amino acids? As we have examined before (Salimijazi et al., 2020), we can improve efficiency by swapping the Calvin cycle for the an alternative CO_2_ fixation cycle (Figure 3). As an aside, the only alternative N_2_-fixation pathway uses the iron-vanadium nitrogenase, that requires 40 ATP and 12 Fd_red_ for each N_2_ fixed (KEGG reaction R12084), compared with 16 ATP and 8 Fd_red_ for the more common iron-molybdenum-cobalt nitrogenase (KEGG reaction R05185).

Not unexpecetedly, the order of efficiency of amino acid synthesis efficiency is approximately the same as the order of efficiency of butanol synthesis. As before (Salimijazi et al., 2020), the 4HB cycle, which performed least well for butanol synthesis (Salimijazi et al., 2020), also performed least well for amino acid synthesis. Likewise, the Wood-Ljungdahl pathway performed the best (Figure 3A).

With increasing efficiency comes decreasing electricity cost (Figure 3B). The average cost of producing a gram of amino acid with H_2_-4HB is $84.3_{-6.8}^{+0.9}kJg^{-1}$ and $63.7_{-5.4}^{+0.7}kJg^{-1}$ with H_2_-WL (costs of 0.07 and 0.05 ¢ g^−1^). Swapping to EEU-4HB increases the costs $97.3_{-15.3}^{+3.3}kJg^{-1}$ , and swapping to EEU-WL reduces them to $70.9_{-9.1}^{2.8}kJg^{-1}$ (costs of 0.08 and 0.06 ¢ g^−1^).

### Oxygen Requirements of Electromicrobial Protein Production Are Low

Using Eqs 13–17 and the cbb_glycine_O2.py code in the electrofoods package, we find that under nominal conditions (Δ*U*_membrane_ = 140 mV), for an H_2_-mediated system using the Calvin cycle, 21.5 *e* ^−^ are needed to synthesize 1 molecule of glycine (supplied by 10.75 H_2_ molecules, or put better, 43 molecules of H_2_ are used to generate 4 glycine molecules). Generating 10.75 molecules of H_2_ by water-splitting co-generates 5.375 molecules of O_2_. However, only 1.875 molecules of O_2_ are actually needed to generate the ATP needed for glycine synthesis. Thus, almost ^2^/_3_rds of the O_2_ generated by water-splitting can be purged from the system to minimize cathode side-reactions.

Likewise, for an EEU-mediated system using the Calvin cycle, 30 *e* ^−^ are needed to generate glycine, releasing 7.5 O_2_ molecules. However, only 4 O_2_ molecules are actually consumed in generating proton motive force. Thus, almost half of the O_2_ generated by water-splitting can be purged from system to minimize cathode side-reactions (**RCv2_1.01**).

### Electromicrobial Protein Is an Energy-Efficient Alternative to Current Protein Production Technologies

How do the upper-limit efficiencies predicted for EMP protein production compare with real world production efficiencies and energy costs? Most rigorous estimates of the total cradle-to-farm gate energy costs needed to produce a gram of beef, chicken, pork, eggs, and dairy (Williams et al., 2006); soybeans (Pimentel, 2009); insects (Van Huis et al., 2013) and cultured meat (Tuomisto and Teixeira de Mattos, 2011) consider only primary energy inputs. Estimates of primary energy input start at 44 kJ g^−1^ for soybeans (Pimentel, 2009) and go up to 273 kJ g^−1^ for beef (Williams et al., 2006) (Supplementary Table S4).

However, traditional estimates of energy input into protein production are not suitable for an apples-to-apples comparison to the numbers calculated in this article. These estimates consider the energy content of feed stocks such as grain and milk; and infrastructural costs such as transportation to the farm gate and tilling land. In the case of soy bean production, the estimates do not include the energy delivered by sunlight to the system to initially fix CO_2_, N_2_ and synthesize amino acids. Likewise, for livestock and dairy production, they do not include the energy content of the sunlight needed to produce the feed, only its final energy content.

Traditional energy input estimates of protein production are not wrong. Quite rightly, sunlight has been thought of as free of cost and global warming concerns. Furthermore, traditional analyses rightly concern themselves with necessary fossil energy inputs. However, as global agricultural production expands, the land for agriculture becomes an increasingly precious commodity. As a result, efficiency of use of sunlight becomes increasingly important.

Likewise, our analysis explicitly ignores infrastructural costs. While we would like to think that bioreactor production of protein could avoid many of these costs, simply thinking this does not make it so. We cannot say so with any certainty if the infrastructure energy costs, such as stirring, heating, gas exchange, are less than the energy inputs associated with agriculture or livestock farming needed to produce a gram of protein.

Estimates of photosynthetic cost of producing protein are the closest comparison point to our work. The closest comparison point to this work is a recent comparison of year round production of protein rich crops, and their protein content with an empirical model of electromicrobial production methods by Leger et al. (2021). The analysis by Leger et al. allows for calculation of the solar energy costs of photosynthetic production (Supplementary Table S5). Energy costs range from 47 MJ g^−1^ (*η*_SP_ = 0.035%) for soybeans grown in the United States to 408 MJ g^−1^ (*η*_SP_ = 0.004%) for maize grown in India (Supplementary Table S5).

In contrast, Leger et al. (2021) estimate an averaged sunlight to protein production efficiency of between 0.29% (minimum food production efficiency) and 0.87% (maximum feed production efficiency) using a solar PV driven Methanol-RUMP pathway. These results presented here suggest that these efficiencies, at least instantaneously could be pushed almost an order of magnitude higher.

## Conclusion

In this work, we examined a fundamental, molecular-scale model of electromicrobial production of amino acids. It is important to re-state here that this calculation does not predict the performance of any naturally-occurring organism. It simply considers a set of redox transformations and enzymatic reactions, and predicts an upper limit efficiency for any natural or synthetic organism using these reactions.

Electromicrobial protein production could address many issues surrounding modern protein production including greenhouse gas emissions (Tuomisto and Teixeira de Mattos, 2011; Garnett, 2014; Smetana et al., 2015), nitrogen run-off, and land use (Carpenter, 2005; Audsley et al., 2010; Tuomisto and Teixeira de Mattos, 2011; Guo et al., 2019; Mishra et al., 2020). Recent results by Leger et al. (2021) suggest that the solar to protein conversion efficiency of agriculture could be improved by an order or magnitude by combining PV with electromicrobial production technologies.

We examined electromicrobial protein production systems that assimilate N_2_ using a FeMo nitrogenase reaction; assimilate carbon using one of the six known natural CO_2_-fixation pathways (3HP/4HB, rTCA, WL, 4HB, CBB, 3HP) pathways or assimilate formic acid with the artificial formolase pathway; and uptake electrons and energy through H_2_-oxidation or extracellular electron uptake. The costs of N_2_-fixation mean that electromicrobial protein production is likely never to be as efficient as carbohydrate electromicrobial production. But, our results suggest that they could approach it.

The least efficient system (EEU coupled with the 4HB cycle; EEU-4HB) required $97.3_{-15.3}^{+3.3}kJg^{-1}$ of an average amino acid (Figure 3B) (corresponds to an electrical to protein energy conversion efficiency, $\eta_{EP}=17.6_{-2.4}^{+0.6}\%$ ; Figure 3A). The most efficient system (H_2_-WL) required only $63.7_{-5.4}^{+0.7}kJg^{-1}$ of amino acids (Figure 3B) ( $\eta_{EP}=26.9_{-2.1}^{+0.3}\%$ , Figure 3A). If supplied with electricity by a perfectly efficient single junction Si PV the EEU-4HB system would produce protein with an efficiency of $\eta_{SP}=5.8\%$ , while the H_2_-WL system would produce protein with an efficiency of $\eta_{SP}=8.9\%$ . These results suggest that the process proposed by Leger et al. (2021) could be improved, at least instantaneously, by another order of magnitude.

What’s the best way to achieve the potential of electromicrobial protein production? All of the systems considered in this study rely upon the presence of at least a small amount (≥a few hundred ppm) O_2_ to generate the maximum amount of reducing equivalents from incoming electrons (Torella et al., 2015; Rowe et al., 2018; Rowe et al., 2021).

Natural options exist for carbon assimilation in high efficiency engineered EMP systems. For carbon assimilation, the Calvin cycle, 3HP cycle, and Formolase pathway can all be operated in the presence of O_2_. In fact, the H_2_-oxidizing microbe *Ralstonia eutropha* (the chassis organism for the Bionic Leaf which uses the Calvin cycle) fixes CO_2_ in the presence of at least 1% O_2_, while the Fe-oxidizing microbe *Sideroxydans lithotrophicus* ES-1 uses EEU to power CO_2_ fixation in a micro-aerobic environment.

However, N_2_-fixation poses a uniquely formidable challenge for high efficiency electromicrobial production. Over the past decade, several groups have incorporated genes for N_2_-fixation into *E. coli* and demonstrated functional N_2_-fixation (Temme et al., 2012; Wang et al., 2013; Li et al., 2016; Yang et al., 2018; Li and Chen, 2020; Ryu et al., 2020). But, despite tantalizing possibilities (MacKellar et al., 2016), all known nitrogenase enzymes are sensitive to O_2_. This creates a fundamental incompatibility between EEU and N_2_-fixation that needs to be solved.

Creation of an O_2_-tolerant nitrogenase may be a tall order for evolution. Unlike other enzymes useful in sustainable energy applications like the hydrogenase (Barstow et al., 2011), there are plenty of evolutionary pressures to drive the creation of an O_2_-tolerant nitrogenase. Despite plenty of demand and opportunities for an O_2_-tolerant nitrogenase to emerge, nature has not presented one.

To date, nature has solved the problem of operating the nitrogenase in an O_2_-rich environment by sequestering it. For example, root nodules in leguminous plants provide an O_2_-shielded environment for symbiotic N_2_-fixing microbes. Likewise filamentous N_2_-fixing cyanobacteria are able to operate the nitrogenase enzyme inside O_2_-impermeable differentiated cells called heterocysts while simultaneously operating oxygenic photosynthesis to generate reducing equivalents in adjacent cells (Bothe et al., 2010). A similar approach, or recent advances in compartmentalization in synthetic biology (Chen and Silver, 2012; Chen et al., 2013; Polka and Silver, 2016; Butterfield et al., 2017; Flamholz et al., 2020), give a menu of options for building a synthetic O_2_-resistant compartment for the nitrogenase. Achieving this goal is likely to represent a major challenge in synthetic biology.

Development of an O_2_-resistant compartment will also enable the implementation of highly efficient CO_2_-fixation pathways like the 3HP/4HB cycle, rTCA cycle and Wood-Ljungdahl pathway in synthetic organisms that simultaneously use O_2_ as a metabolic terminal electron acceptor.

Failure to operate enzymatic N_2_-fixation does not spell the end of the road for electromicrobial protein production however. Much as there has been significant development of electrochemical CO_2_ reduction to C_1_ compounds, recent developments in electrochemical N_2_ reduction to ammonia could be a promising complement to biological production of complex amino acids (Guo et al., 2019).

## Data Availability

The original contributions presented in the study are included in the article/Supplementary Material, further inquiries can be directed to the corresponding author. All code used in calculations in this article is available at https://github.com/barstowlab/electrofoods and is archived on Zenodo at https://doi.org/doi:10.5281/zenodo.5847529.
