# Supplementary material for: Thermodynamic Constraints on Electromicrobial Protein Production
Source: Front Bioeng Biotechnol. 2022 Feb 21;10:820384. doi: 10.3389/fbioe.2022.820384 (PMC8899463; doi:10.3389/fbioe.2022.820384)
Supplement: Supplementary file 1 [file DataSheet3.docx]

Supplementary Information for:

Thermodynamic Constraints on Electromicrobial Protein Production

Lucas Wise^1^, Sabrina Marecos^2*^, Katie Randolph^2*^, Mohamed Hassan^2^, Eric Nshimyumukiza^2^, Jacob Strouse^2^, Farshid Salimijazi^2^, and Buz Barstow^2†^

^1^Department of Food Sciences, Cornell University, Ithaca, NY 14853, USA

^2^Department of Biological and Environmental Engineering, Cornell University, Ithaca, NY 14853, USA

^*^These authors contributed equally to this article.

†Corresponding author:

Buz Barstow, 228 Riley-Robb Hall, Cornell University, Ithaca, NY 14853; bmb35@cornell.edu

# Supplementary Information Tables

**Table S1.** Symbols used in this article.

**Table S2.** Molecular weights and energy densities for products considered in this article.

**Table S3.** Carbon-fixation and -assimilation, and nitrogen-fixation reactions considered in this article.

**Table S4.** Primary energy inputs for representative protein sources.

**Table S5.** Solar energy costs of photosynthetic protein production.

# Supplementary Information Datasets

**Dataset S1.** Enzymatic reactions for synthesis of amino acids and D-glucose from metabolic intermediates.

**Dataset S2.** Net molecular input requirements for product synthesis production by 6 naturally-occurring CO_2_-fixation cycles and the synthetic Formolase formate assimilation pathway.

| **Symbol** | **Unit** | **Description** |
| --- | --- | --- |
| *E*_protein_ | J molecule^-1^ | Energy carried per amino acid or protein molecule. |
| *Ṅ*_protein_ | molecule s^-1^ | Amino acid (or protein) molecules produced per second by electromicrobial production system. |
| *N*_A_ | molecule Mol^-1^ | Avogadro constant. |
| F | A s Mol^-1^ | Faraday Constant |
| *P*_e, total_ | J s^-1^ | Total electrical power input into electromicrobial production system. |
| *M*_protein_ | g Mol ^-1^ | Molecular weight of the protein molecule |
| *e* | A s | Fundamental charge |
| *ν*_ep_ | # | Number of electrons needed for synthesis of an amino acid molecule |
| Δ*U*_cell_ | V | Potential difference across bio-electrochemical cell |
| *ν*_e, add_ | # | Number of electrons needed to convert a C_1_ compound to a protein product |
| *ν*_r_ | # | Number of primary reduction products to make a molecule of final product |
| *ν*_er_ | # | Number of electrons to reduce CO_2_ to a primary reduction product |
| *ν*_Cr_ | # | Number of carbon atoms per primary reduction product |
| *ξ*_I2_ | # | Faradaic efficiency of the bio-electrochemical cell |
| *ξ*_I1_ | # | Faradaic efficiency of the primary abiotic cell |
| *ξ*_C_ | # | Carbon transfer efficiency from cell 1 to cell 2 |
| *ν*_p, NADH_ | # | Number of NAD(P)H molecules needed to make a final protein molecule |
| *ν*_p, Fd_ | # | Number of Fd molecules needed to make a final protein molecule |
| *ν*_p, ATP_ | # | Number of ATP molecules needed to make a final protein molecule |
| Δ*G*_ATP/ADP_ | J | Free energy for regeneration of ATP |
| Δ*U*_membrane_ | V | Inner membrane potential difference |
| U_H2_ | V | Standard potential of proton reduction to H_2_ |
| *U*_acceptor_ | V | Standard potential of terminal electron acceptor reduction |
| *U*_Q_ | V | Redox potential of the inner membrane electron carrier |
| *U*_NADH_ | V | Standard potential of NADH |
| *U*_Fd_ | V | Standard potential of Ferredoxin |
| *C*_EP_ | J g^-1^ | Electrical energy cost per unit mass |
| *C*_SP_ | ¢ g^-1^ | Financial cost efficiency per unit mass |
| *η*_EP_ | % | Electrical to product (*e.g.,* protein) energy conversion efficiency |
| *η_S_*_P_ | % | Solar to product (*e.g.,* protein) energy conversion efficiency |
| $\nu_{O_{2}\mathrm{evolved},(H_{2}\vert\mathrm{EEU})}$ | # | Number of O_2_ molecules evolved in either H_2_- or EEU-mediated EMP |
| $\nu_{O_{2}\mathrm{consumed},(H_{2}\vert\mathrm{EEU})}$ | # | Number of O_2_ molecules consumed in either H_2_- or EEU-mediated EMP |

**Table S1.** Symbols used in this article.

| Amino Acid | Symbol | Molecular Weight (Da) | Energy Density (kJ g^-1^) | Energy Density (J molecule^-1^) | Molecular Formula | Carbons per Molecule |
| --- | --- | --- | --- | --- | --- | --- |
| Alanine | ALA | 89 | 13.98 | 2.066E-18 | C_3_H_7_NO_2_ | 3 |
| Arginine | ARG | 174 | 13.58 | 3.924E-18 | C_6_H_14_N4O_2_ | 6 |
| Asparagine | ASN | 132 | 9.38 | 2.056E-18 | C_4_H_8_N_2_O3 | 4 |
| Aspartate | ASP | 133 | 9.23 | 2.039E-18 | C_4_H_7_NO_4_ | 4 |
| Cysteine | CYS | 121 | 17.44 | 3.504E-18 | C_3_H_7_NO_2_S | 3 |
| Glutamine | GLN | 146 | 12.69 | 3.077E-18 | C_5_H_10_N_2_O_3_ | 5 |
| Glutamate | GLU | 147 | 12.53 | 3.059E-18 | C_5_H_9_NO_4_ | 5 |
| Glycine | GLY | 75 | 8.67 | 1.080E-18 | C_2_H_5_NO_2_ | 2 |
| Histidine | HIS | 155 | 13.57 | 3.493E-18 | C_6_H_9_N_3_O_2_ | 6 |
| Isoleucine | ILE | 131 | 23.80 | 5.177E-18 | C_6_H_13_NO_2_ | 6 |
| Leucine | LEU | 131 | 23.80 | 5.177E-18 | C_6_H_13_NO_2_ | 6 |
| Lysine | LYS | 146 | 20.02 | 4.854E-18 | C_6_H_14_N_2_O_2_ | 6 |
| Methionine | MET | 149 | 21.41 | 5.297E-18 | C_5_H_11_NO_2_S | 5 |
| Phenylalanine | PHE | 165 | 25.06 | 6.866E-18 | C_9_H_11_NO_2_ | 9 |
| Proline | PRO | 115 | 20.10 | 2.039E-18 | C_5_H_9_NO_2_ | 5 |
| Serine | SER | 105 | 10.36 | 1.806E-18 | C_3_H_7_NO_3_ | 3 |
| Threonine | THR | 119 | 13.94 | 2.755E-18 | C_4_H_9_NO_3_ | 4 |
| Tryptophan | TRP | 204 | 23.39 | 7.924E-18 | C_11_H_12_N_2_O_2_ | 11 |
| Tyrosine | TYR | 181 | 21.77 | 6.543E-18 | C_9_H_11_NO_3_ | 9 |
| Valine | VAL | 117 | 21.27 | 4.132E-18 | C_5_H_11_NO_2_ | 5 |
| Average Amino Acid | AVE | 136.75 | 16.7995 | 3.815E-18 | - | 5 |
| Glucose | GLUC | 180.16 | 15.55 | 4.652E-18 | C_6_H_12_O_6_ | 6 |
| Butanol | BUT | 74.12 | 36.6 | 4.505E-18 | C_4_H_10_O | 4 |

**Table S2.** Molecular weights and energy densities for products considered in this article. Data for amino acids from May *et al*. [May1990a].

| Reaction | Reference |
| --- | --- |
| ***1. Calvin Cycle*** | |
| 2 CO_2_ + 7 ATP + 4 NADH → 1 Acetyl-CoA | Salimijazi *et al*. [Salimijazi2020b]. |
| 3 CO_2_ + 7 ATP + 5 NADH → 1 Pyruvate | Salimijazi *et al* [Salimijazi2020b]. |
| ***2. Wood-Ljungdahl Pathway*** | |
| 4 CO_2_ + 2 ATP + 8 NADH → 2 Acetyl-CoA | Berg [Berg2011a]. |
| 2 Fd_red_ + Acetyl-CoA + CO_2_ → Pyruvate | KEGG R01196. |
| ***3. Reductive TCA Cycle*** |  |
| 4 CO2 + 4 ATP + 8 NADH → 2 Acetyl-CoA | Alissandratos *et al.* [Alissandratos2015a], Claassens *et al*. [Claassens2016a]. |
| 2 Fd_red_ + Acetyl-CoA + CO_2_ → Pyruvate | KEGG R01196. |
| ***4. 3-hydroxypropionate/4-hydroxybutyrate Cycle*** | |
| 6 HCO_3_^-^ + 10 ATP + 10 NADH → 2 pyruvate | Berg *et al*. [BergI2007a], Claassens *et al*. [Claassens2016a]. |
| 2 Pyruvate → 2 Acetyl-CoA + 2 NADH + 2 CO_2_ | Berg [Berg2002a], Schomburg *et al.* [Schomburg2017a]. |
| ***5. 3-hydroxypropionate Cycle*** |  |
| 6 HCO_3_^-^ + 10 ATP + 12 NADH → 2 Pyruvate | Zarzycki *et al*. [Zarzycki2009a], Herter *et al*. [Herter2002a], Berg [Berg2002a]. |
| 2 Pyruvate → 2 Acetyl-CoA + 2 NADH + 2 CO_2_ | Zarzycki *et al*. [Zarzycki2009a], Herter *et al*. [Herter2002a], Berg [Berg2002a]. |
| ***6. 4-hydroxybutyrate Cycle*** | |
| 1 CO_2_ + 1 HCO_3_^-^ + 3 ATP + 1 NADH + 6 Fd_red_ → 1 Acetyl-CoA | Huber *et al*. [Huber2008a]. |
| 2 Pyruvate → 2 Acetyl-CoA + 2 NADH + 2 CO_2_ | Berg [Berg2002a], Schomburg *et al.* [Schomburg2017a]. |
| ***7. Formolase Pathway*** | |
| 6 HCO_2_^-^ + 10 ATP + 4 NADH → 2 3-PG | Siegel *et al*. [Siegel2015a], Bar-Even *et al*. [Bar-Even2016a]. |
| 2 3-PG → 2 Pyruvate + 2 ATP | Berg [Berg2002a]. |
| 2 Pyruvate → 2 Acetyl-CoA + 2 NADH + 2 CO_2_ | Berg [Berg2002a], Schomburg *et al.* [Schomburg2017a]. |

**Table S3.** CO_2_-fixation and C_1_-assimilation reactions. CO_2_-fixation and C_1_-assimilation reactions considered in this article were first assembled in Salimijazi *et al*. [Salimijazi2020b] and are restated here for convenience. Overall reactions for production of metabolic intermediates by 6 naturally-occurring CO_2_-fixation cycles and the synthetic Formolase formate assimilation pathway, and the FeMoCo nitrogenase N_2_-fixation reaction. Reactions can be referenced KEGG database [Kanehisa2000a, Kanehisa2019a, Kanehisa2021a]. Fd_red_: Reduced Ferredoxin; 3-PG: 3-Phosphoglycerate.

| Food Source | Energy Consumption (MJ kg^-1^ protein) | References | Notes |
| --- | --- | --- | --- |
| Beef | 187 - 273 | Williams *et al.* [Williams2006a] | All selected LCAs use a “Cradle to Farm/Factory-Gate” strategy for assessing energy use. This means no transport, retail or household considerations for the most apples-to-apples comparison possible. These numbers are quite variable depending on the inputs per LCA. |
| Pork | 119 - 129 | Williams *et al.* [Williams2006a] |  |
| Chicken | 80 - 96 | Williams *et al.* [Williams2006a] |  |
| Eggs | 87 - 95 | Williams *et al.* [Williams2006a] |  |
| Soybeans | 44.22 | Pimentel *et al.* [Pimentel2009a] | Solar energy input is not considered. |
| Milk/Dairy | 67 - 68 | Williams *et al.* [Williams2006a] |  |
| Insect | 170 | Oonincx *et al.* [Oonincx2012a] | This is an example where MJ kg^-1^ food and MJ kg^-1^ protein makes a huge difference. |
| Cultured Meat | 131.9 - 166.5 | Tuomisto *et al.* [Tuomisto2011a] | Divided by protein content of 19.1% as per reference (similar to lean meat protein %) |

**Table S4.** Primary energy inputs for representative protein sources.

| Crop | Country | Protein Produced (g m^-2^ yr^-1^) | Energy Stored in Protein (kJ m^-2^ yr^-1^) | Median Irradiance kJ m^-2^ yr^-1^ | Solar Energy Cost, *C*_SP_ (kJ g^-1^) | Solar to Protein Energy Conversion Efficiency, *η*_SP_ (%) | Solar to Protein Energy Conversion Efficiency Error (%) |
| --- | --- | --- | --- | --- | --- | --- | --- |
| Maize | USA | 93 | 1,556 | 5,684,400 | 61,123 | 0.027% | 0.003% |
| Maize | Bulgaria | 63 | 1,050 | 5,061,600 | 80,343 | 0.021% | 0.005% |
| Maize | India | 22 | 372 | 8,967,600 | 407,618 | 0.004% | 0.001% |
| Maize irrigated | USA | 118 | 1,975 | 6,130,800 | 51,956 | 0.032% | 0.002% |
| Maize sillage | USA | 73 | 1,218 | 5,194,800 | 71,162 | 0.023% | 0.003% |
| Sugar beet | USA | 99 | 1,648 | 5,400,000 | 54,545 | 0.030% | 0.003% |
| Soybean | USA | 120 | 2,004 | 5,677,200 | 47,310 | 0.035% | 0.005% |

**Table S5.** Solar energy inputs for protein production by crops. Adapted from Table S1G in Leger *et al*. [Leger2021a]. Solar energy costs per gram of protein were added by us, and units were converted to match those in our article.

# Supplementary Information References

[Alissandratos2015a] A. Alissandratos and C. J. Easton. “Biocatalysis for the application of CO_2_ as a chemical feedstock”. *Beilstein Journal of Organic Chemistry* 11 (2015), pp. 2370–2387. [doi:10.3762/bjoc.11.259](https://doi.org/10.3762/bjoc.11.259).

[Bar-Even2016a] A. Bar-Even. “Formate Assimilation: The Metabolic Architecture of Natural and Synthetic Pathways.” *Biochemistry* 55 (2016), pp. 3851–63. [doi:10.1021/acs.biochem.6b00495](https://doi.org/10.1021/acs.biochem.6b00495).

[Barstow2021a] B. Barstow. Electrofoods. [doi:10.5281/zenodo.5805345](https://doi.org/10.5281/zenodo.5805345).

[Berg2002a] J. Berg, J. Tymoczko, and L. Stryer. Biochemistry. 5th. New York, NY: W H Freeman, 2002.

[Berg2011a] I. A. Berg. “Ecological Aspects of the Distribution of Different Autotrophic CO_2_ Fixation Pathways”. *Applied and Environmental Microbiology* 77 (2011), pp. 1925–1936. [doi:10.1128/aem.02473-10](https://doi.org/10.1128/aem.02473-10).

[BergI2007a] I. A. Berg, D. Kockelkorn, W. Buckel, and G. Fuchs. “A 3-Hydroxypropionate/4-Hydroxybutyrate Autotrophic Carbon Dioxide Assimilation Pathway in Archaea”. *Science* 318 (2007), pp. 1782–1786. [doi:10.1126/science.1149976](https://doi.org/10.1126/science.1149976).

[Claassens2016a] N. J. Claassens, D. Z. Sousa, V. A. M. dos Santos, W. M. de Vos, and J. van der Oost. “Harnessing the power of microbial autotrophy”. *Nature Reviews Microbiology* 14 (2016), pp. 692–706. [doi:10.1038/nrmicro.2016.130](https://doi.org/10.1038/nrmicro.2016.130).

[Herter2002a] S. Herter, G. Fuchs, A. Bacher, and W. Eisenreich. “A bicyclic autotrophic CO_2_ fixation pathway in *Chloroflexus aurantiacus*”. *Journal of Biological Chemistry* 277 (2002), pp. 20277–20283. [doi:10.1074/jbc.m201030200](https://doi.org/10.1074/jbc.m201030200).

[Huber2008a] H. Huber, M. Gallenberger, U. Jahn, E. Eylert, I. A. Berg, D. Kockelkorn, W. Eisenreich, and G. Fuchs. “A dicarboxylate/4-hydroxybutyrate autotrophic carbon assimilation cycle in the hyperthermophilic *Archaeum Ignicoccus hospitalis*”. *Proceedings of the National Academy of Sciences* 105 (2008), pp. 7851–7856. [doi:10.1073/pnas.0801043105](https://doi.org/10.1073/pnas.0801043105).

[Kanehisa2000a] M. Kanehisa and S. Goto. “KEGG: Kyoto Encyclopedia of Genes and Genomes”. *Nucleic Acids Research* 28.1 (2000), pp. 27–30. [doi:10.1093/nar/28.1.27](https://doi.org/10.1093/nar/28.1.27).

[Kanehisa2019a] M. Kanehisa. “Toward understanding the origin and evolution of cellular organisms”. Protein *Science* 28.11 (2019), pp. 1947–1951. [doi:10.1002/pro.3715](https://doi.org/10.1002/pro.3715).

[Kanehisa2021a] M. Kanehisa, M. Furumichi, Y. Sato, M. Ishiguro-Watanabe,and M. Tanabe. “KEGG: integrating viruses and cellular organisms”. *Nucleic Acids Research* 49.D1 (2020), gkaa970–. [doi:10.1093/nar/gkaa970](https://doi.org/10.1093/nar/gkaa970).

[Leger2021a] D. Leger, S. Matassa, E. Noor, A. Shepon, R. Milo, and A. Bar-Even. “Photovoltaic-driven microbial protein production can use land and sunlight more efficiently than conventional crops”. *Proceedings of the National Academy of Sciences* 118.26 (2021), e2015025118. [doi:10.1073/pnas.2015025118](https://doi.org/10.1073/pnas.2015025118).

[May1990a] M.E. May, and J.O. Hill. “Energy content of diets of variable amino acid composition”. *Am J Clin Nutrition* 52, 770–776 (1990). [doi:10.1093/ajcn/52.5.770](https://doi.org/10.1093/ajcn/52.5.770).

[Pimentel2009a] D. Pimentel. “Energy Inputs in Food Crop Production in Developing and Developed Nations”. *Energies* 2.1 (2009), pp. 1–24. [doi:10.3390/en20100001](https://doi.org/10.3390/en20100001).

[Salimijazi2020b] F. Salimijazi, J. Kim, A. M. Schmitz, R. Grenville, A. Bocarsly, and B. Barstow. “Constraints on the Efficiency of Engineered Electromicrobial Production”. *Joule* 4 (2020), pp. 2101–2130. [doi:10.1016/j.joule.2020.08.010](https://doi.org/10.1016/j.joule.2020.08.010).

[Schomburg2017a] I. Schomburg, L. Jeske, M. Ulbrich, S. Placzek, A. Chang,and D. Schomburg. “The BRENDA enzyme information system–From a database to an expert system”. *Journal of Biotechnology* 261 (2017), pp. 194–206. [doi:10.1016/j.jbiotec.2017.04.020](https://doi.org/10.1016/j.jbiotec.2017.04.020).

[Siegel2015a] J. B. Siegel, A. L. Smith, S. Poust, A. J. Wargacki, A.Bar-Even, C. Louw, B. W. Shen, C. B. Eiben, H. M.Tran, E. Noor, J. L. Gallaher, J. Bale, Y. Yoshikuni, M. H. Gelb, J. D. Keasling, B. L. Stoddard, M. E. Lidstrom, and D. Baker. “Computational protein design enables a novel one-carbon assimilation pathway”. *Proceedings of the National Academy of Sciences* 112 (2015), p. 3704-3709. [doi:10.1073/pnas.1500545112](https://doi.org/10.1073/pnas.1500545112).

[Tuomisto2011a] H. L. Tuomisto and M. J. T. d. Mattos. “Environmental Impacts of Cultured Meat Production”. *Environmental Science & Technology* 45.14 (2011), pp. 6117–6123. [doi:10.1021/es200130u](https://doi.org/10.1021/es200130u).

[Williams2006a] A. Williams, E. Audsley, and D. Sandars. “Determining the environmental burdens and resource use in the production of agricultural and horticultural commodities. Defra project report IS0205”. Tech. rep. Cranfield University and Defra, 2006.

[Zarzycki2009a] J. Zarzycki, V. Brecht, M. Müller, and G. Fuchs. “Identifying the missing steps of the autotrophic 3-hydroxypropionate CO_2_ fixation cycle in *Chloroflexus aurantiacus*”. *Proceedings of the National Academy of Sciences* 106 (2009), p. 21317. [doi.10.1073/pnas.0908356106](https://doi.org/10.1073/pnas.0908356106).
